# Phages as potential life-saving therapeutic option in the treatment of multidrug-resistant urinary tract infections

**DOI:** 10.3389/abp.2025.14264

**Published:** 2025-02-11

**Authors:** Beata Zalewska-Piątek, Michalina Nagórka

**Affiliations:** Department of Biotechnology and Microbiology, Chemical Faculty, Gdańsk University of Technology, Gdańsk, Poland

**Keywords:** urinary tract infections, phages, phage therapy, antimicrobial resistance, multidrug-resistant uropathogens

## Abstract

Urinary tract infections (UTIs) are among the most common bacterial infections worldwide and increasing antimicrobial resistance (AMR) challenges conventional antibiotic treatments. Phage therapy (PT) has emerged as a promising alternative due to its specificity, safety and efficacy against multidrug-resistant (MDR) pathogens causing infectious diseases. PT demonstrates significant potential in treating chronic and recurrent UTIs, also including catheter-associated infection by reducing bacterial biofilms, delaying catheter blockage, and enhancing antibiotic efficacy when used in combination. Clinical trials and case studies have reported high rates of bacterial eradication and symptom improvement with minimal side effects. Although endotoxin release and immune activation during treatment should continue to be investigated. The aim of this review is to present issues related to the use of phages in the treatment of UTIs of various etiological origins in selected patients, including those with comorbidities, taking into account the legal regulations, safety and effectiveness of this experimental therapy. The growing prevalence of MDR uropathogens highlights the urgent need for alternative therapies, such as those based on phages in order to treat antibiotic-resistant infections and improve patient outcomes. Despite the great potential of PT, its clinical implementation and use of phages as a routine treatment for bacterial infections requires rigorous trials, standardized production protocols and regulatory advancements.

## Introduction

Urinary tract infections (UTIs) are among the most commonly treated infections in the world accounting for approximately 150 million cases per year (Foxman, 2014; Leitner et al., 2024). These infections lead to significant increases in healthcare costs, given that more than 50% of women experience at least one UTI episode through their lifetime and in 20%–40% of patients the infection recurs within a few months (Aslam et al., 2020; Zalewska-Piątek and Piątek, 2020). Complicated catheter-related UTIs (CAUTIs) are of particular concern to healthcare facilities (Kranz et al., 2024). Furthermore, approximately 75% of hospital-acquired UTIs are associated with urinary catheter, assuming that 15%–25% of patients are catheterized during their hospital stay (Werneburg, 2022; Centers for Disease Control and Prevention, CDC, 2024).

The urological diseases are very often caused by uropathogenic strains of *Escherichia coli* (UPEC) which are the major bacterial pathogen causing 75%–85% of uncomplicated and 50%–65% of complicated UTIs, respectively (Table 1). In addition to UPEC, uropathogens contributing to UTIs also include *Klebsiella pneumoniae*, *Staphylococcus saprophyticus*, *Enterococcus faecalis*, *group B Streptococcus, Proteus mirabilis*, *Pseudomonas aeruginosa* and *Staphylococcus aureus* (Flores-Mireles et al., 2015; Zalewska-Piątek et al., 2019; Mijbel Ali et al., 2021). Increasing rates of antimicrobial resistance (AMR) among uropathogens to commonly used antibiotics have provided the impetus to consider alternative treatment strategies, including phage-based therapy (Kot, 2019; Guo et al., 2021; Murray et al., 2022). Such therapeutic interventions allow UTIs to be controlled. They also limit the development of chronic and recurrent urological diseases especially in patients with urinary tract dysfunction (Zaitsev et al., 2024).

**TABLE 1:** Brief description of urinary tract infections (UTIs) (Hooton, 2012; Levison and Kaye, 2013; Lo et al., 2014; Flores-Mireles et al., 2015; Johnson and Russo, 2018; Zalewska-Piątek et al., 2019; Zalewska-Piątek and Piątek, 2020; Belyayeva et al., 2024).

Classification of UTIs	
Source of infection	
Acquired by the community or in hospitals	
Related to healthcare	
Clinical picture and severity	
The upper UTIs	
Pyelonephritis	Infection and inflammation of renal pelvis and kidneys Nature of the disease acute, mild or moderate
Urosepsis	Sepsis (severe infection) Septic shock
The lower UTIs	
Cystitis	Infection and inflammation of the bladder
Risk factors and predisposition	
Uncomplicated UTIs	No anatomical defect of the UT No UT instrumentation No pregnancy
Complicated UTIs	Renal failure and transplantation Anatomical UT abnormalities (retention, urinary obstruction) Indwelling catheters, medical drainage devices Immunosuppression Previous antibiotic exposure Pregnancy

UPEC strains have developed various mechanisms that determine their survival in the urinary tract environment, chronicity and recurrence of infections. The most important bacterial defense systems include adhesion to the uroepithelium (a key step in pathogenesis) and invasion of the superficial umbrella cells, formation of biofilm and the metabolically active biofilm-like intracellular bacterial communities (IBCs), and establishment of quiescent intracellular reservoirs (QIRs) in the bladder. OIR structures consist of only 4–10 non-replicating bacterial cells within F-actin membrane-bound compartments that are capable of surviving for several months and can be a potential source of recurrent infection (Flores-Mireles et al., 2015; Zalewska-Piątek and Piątek, 2020). Therefore, phage therapy based on phage cocktails and combined phage-antibiotic therapy may represent a turning point in the treatment of UTIs, their prevention and limitation of further spread of MDR UPEC in the urinary tract and also biofilm formation (Valério et al., 2017; Chegini et al., 2021). However, this requires further studies and a series of clinical trials.

Bacteriophages were discovered independently by Frederick Twort and Félix d’Herelle in 1915 and 1917, respectively (Twort, 1915; D’Herelle, 1917; D’Herelle, 2007). Research into the use of phages as potential therapeutic agents began in the 1910s but was largely forgotten in the antibiotic era during World War II (WWII) (Lin et al., 2017). Phages are bacterial viruses that specifically select and kill bacterial hosts without affecting the human commensal microflora compared to antibiotics. Therefore, “good” commensal bacteria, which control the body’s vital functions, are not attacked by phages. However, during antibiotic therapy, there is no such selection and both “good” and pathogenic bacteria are destroyed (Zalewska-Piątek, 2023).

The development of phage therapy (PT) for the treatment of infectious diseases requires the successful completion of extensive clinical trials according to the guidelines from the U.S. Food and Drug Administration (FDA) and the European Medicines Agency (EMA). Patients infected with multidrug-resistant (MDR) bacteria and for whom antibiotic therapy has failed are treated under an emergency expanded access program for Investigational New Drugs (INDs) approved by the U.S. FDA and EMA (Luong et al., 2020). Since phages are considered by regulatory authorities as biological agents, they must be governed by the guidelines for the Biological Medicinal Products for European trials and the guidelines of the Division of Vaccines and Related Products Application in the U.S.A. (Parracho et al., 2012). The first regulated efficacy trial of PT occurred in 2009 when a phase I clinical trial (approved by the FDA) evaluated the efficacy and safety of a phage cocktail targeting *S. aureus*, *P. aeruginosa* and *E. coli* in venous ulcers (Rhoads et al., 2009).

**FIGURE 1:**
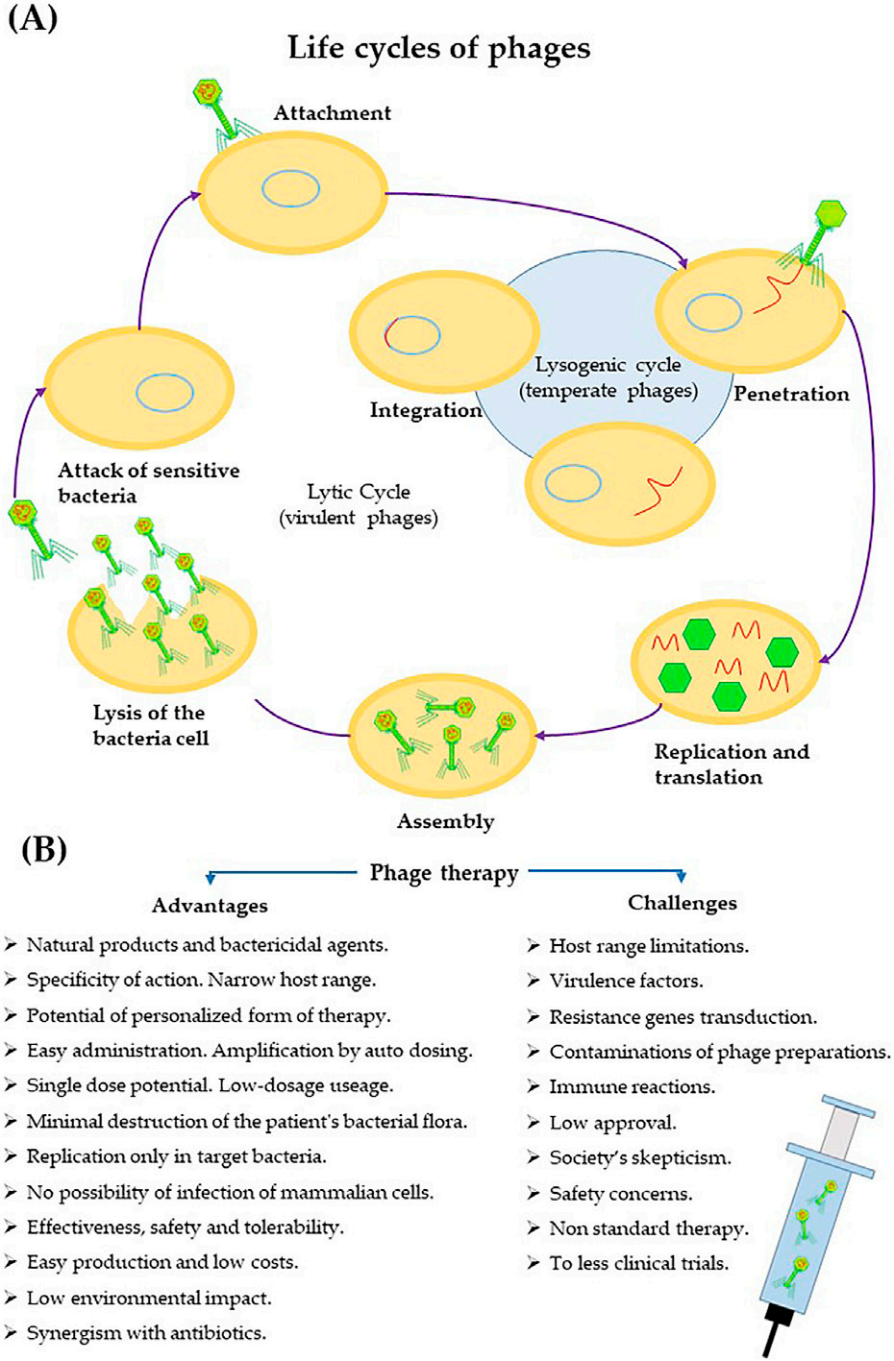
Mechanism of phage action in the context of their use in the treatment of bacterial infections. **(A)** Phage replication cycles. **(B)** Positive aspects of phage therapy (Principi et al., 2019; Zalewska-Piątek and Piątek, 2020; Roughgarden, 2024; Mishra et al., 2024).

Phages, considering their replication cycles, can be divided into lytic and lysogenic viruses (Roughgarden, 2024). The lytic cycle is associated with the lysis of bacterial cells after the production of progeny phages. In turn, the lysogenic cycle is accompanied by the integration of the phage genome into the bacterial genome (prophage state) and replication with it. Under certain environmental conditions, induction can occur, which is associated with the excision of the prophage genome and initiation of the lytic cycle (Figure 1) (Zalewska-Piątek and Piątek, 2020; Roughgarden, 2024). PT is most often based on the use of lytic or genetically modified phages and phage proteins. Phages do not multiply in eukaryotic cells. Therefore, an exponential increase in the number of viral particles is observed once they reach the site of bacterial infection. In turn, when killing bacteria, the removal of phages from the body can be observed. Ultimately, the phages are excreted in the urine by the patient’s kidneys, depending on their size (Ling et al., 2022; Nang et al., 2023).

Antibiotics, discovered in the 1930s (the “miracle drug” penicillin, 1928), became the basis for treating bacterial infections that plague humanity (Fleming, 2001). At the beginning, they showed high therapeutic efficacy. In times of intensive production and effective use of antibiotics, few scientists allowed the possibility of a return to phage-based therapeutic trials. However, soon, due to their widespread use, the rapid emergence of antibiotics-resistant bacteria occurring worldwide was also observed. The United Nations General Assembly in 2016, identified the problem of antimicrobial resistance (AMR) as the “greatest and most urgent global risk” (United Nations, UN, 2016). MDR bacteria such as methicillin-resistant *S. aureus* (MRSA), vancomycin-resistant *Enterococci* (VRE) and extended-spectrum β-lactamase (ESBL) producing *Enterobacteriaceae* represent significant challenges to healthcare systems worldwide. This highlights the need to search innovative forms of treatment for MDR and extensively drug-resistant (XDR) bacterial infections (Ling et al., 2015; Douglas et al., 2023).

In this context, phages are currently considered one of the best alternatives in treating various chronic and recurrent diseases caused by bacteria (Malik et al., 2019; Mijbel Ali et al., 2021). PT is also much safer for the body than therapy based on antibiotics and well tolerated by patients, side effects are rare and not very bothersome (Liu et al., 2021; Strathdee et al., 2023).

## Potential applications of phages in the treatment of UTIs based on selected examples

Phages have been used in medicine as early as 1919, to treat infections caused by *Shigella dysenteriae*, 10 years before the discovery of penicillin (Chanishvili, 2012). The use of phages as therapeutic agents in the treatment of antibiotic-resistant bacterial infections was continued in Europe, America, and Asia until the outbreak of WWII and the expansion of antibiotics in the world. This form of treatment developed rapidly and vigorously. Pharmaceutical companies produced useful phage preparations. In turn, after WWII, PT was gradually replaced by antibiotic therapy as an easy-to-use and effective form of treatment for bacterial infectious diseases. The role of PT and the use of phages as therapeutic agents began to grow again in the 1980s due to the increasing incidence of antibiotic-resistant bacterial strains, especially in hospital wards (Sulakvelidze et al., 2001; Reuter and Kruger, 2020).

UTIs are a very serious problem worldwide. Increasing antibiotic resistance of uropathogens and limited therapeutic options have led to the search of alternative therapies to UTIs, including PT. Furthermore, CAUTIs remain a critical global health challenge with an estimated 150–250 million cases annually. This is the most common type of hospital-acquired infection, which is a major health concern due to the frequent recurrence and complications. CAUTIs are often caused by *E. coli* and *Proteus mirabilis* (Jacobsen et al., 2008). These infections are particularly problematic for individuals undergoing long-term catheterization. Other risk factors that contribute to the development of CAUTIs include female gender, patient age and diabetes (Chenoweth et al., 2014).

*Proteus mirabilis* strains form biofilms that lead to encrustation and blockage of catheters. Such complications can cause urinary reflux, kidney infections, and even life-threatening conditions like sepsis (Wasfi et al., 2020). One of the research teams explored an innovative dual-layered coating system (impregnated with a therapeutic dose of phage released in response to increased urine pH) for catheters. This system was designed to release phages in response to elevated urinary pH specific to *P. mirabilis* infection. Coated catheters doubled the time to blockage compared to uncoated ones, delaying obstruction from 13 to 26 h in a controlled bladder model. Additionally, bacterial populations were significantly reduced, with a remarkable six-log decrease in *P. mirabilis* concentration within 2 hours of activation. This study underscores the potential of PT as a targeted, antibiotic-free treatment for CAUTIs. This technology delays catheter clogging and effectively reduces bacterial biofilms, offering promising opportunities to improve patient outcomes and reduce the healthcare burden associated with long-term catheter use (Milo et al., 2016).

In turn, UPEC strains, as one of the main causes of CAUTIs are a particular problem in patients with spinal cord injuries (Manack et al., 2011). In one study, researchers showed that a cocktail of six phages lysed 82% of the *E. coli* strains tested and reduced biofilm viability by up to 99.5% in tryptic soy broth and 94% in human urine. The phage preparation was effective against young and mature biofilms, including those on silicone catheter materials. Combining the phage cocktail with antibiotics such as trimethoprim/sulfamethoxazole or ciprofloxacin further increased bacterial killing, reduced resistance, and improved efficacy. Through this research, it was also possible to identify the phage-produced depolymerases that contribute to biofilm degradation (Sanchez et al., 2022). These studies highlight the therapeutic potential of phage cocktails for the treatment of MDR CAUTIs. In addition, further evaluation of these treatments on animal models and preparation for clinical application is also planned.

In addition, the first results from the largest clinical trial to date on the treatment of UTIs with phages were recently published. The ELIMINATE trial was aimed at testing the efficacy of phages to treat infections caused by UPEC resistant to antimicrobial drugs. The obtained *in vitro* data showed that Locus’s six-strain phage cocktail was effective against 94% of clinical isolates of *E. coli* analyzed. This study also determined the dosage that will be used in the second round of testing in 288 people (Suh et al., 2024).

Lia et al. (2024) reported the successful treatment of chronic bacterial prostatitis (CBP) in a 65-year-old male using a combination of bacteriophage and antibiotic therapy. CBP infection caused by MDR *E. coli* persisted despite 5 months of antibiotic treatment with sulfamethoxazole/trimethoprim. The patient was treated with customized PT, administered orally and rectally, alongside antibiotics. After several cycles of therapy, the infection was eliminated, as confirmed by negative urine culture results, and the patient had no symptoms or side effects for over a year. This case underscores the potential of personalized PT as a promising tool against chronic, drug-resistant infections (Lia et al., 2024).

Furthermore, Al-Anany et al. (2023) analyzed 55 studies of the effectiveness of PT for UTIs. Clinical and microbiological improvement was shown to occur in more than 72% of cases, although many studies were based on case reports with limited controlled studies. This analysis reinforced the promise of PT as a viable alternative for antibiotic-recurrent UTIs (rUTIs) while also highlighting the need for rigorous trials to confirm efficacy and standardize treatment protocols. These findings emphasize PT’s broad potential and the pressing need to develop it as a therapeutic option for resistant infections (Al-Anany et al., 2023).

In another case, Terwilliger et al. (2021) investigated the effect of a unique phage cocktail to treat a liver transplant patient suffering from rUTIs caused by ESBL-producing *E. coli*. Despite multiple prior antibiotic treatments, the infection persisted, prompting the team to administer a tailored phage cocktail in combination with antibiotics. The therapy managed to control the infection, with the patient experiencing symptom relief and no side effects. Although recurrence of asymptomatic, low-level bacteriuria occurred after treatment, no further antibiotic administration was required, suggesting that PT can achieve microbiological stabilization without complete bacterial eradication. This case illustrates the adaptability of PT as a targeted solution for complex infections and highlights how it can work in conjunction with antibiotics to combat resistant pathogens (Terwilliger et al., 2021).

Furthermore, Johri et al. (2021), described the impact of applied PT to treat a patient with CBP, a condition resistant to conventional antibiotics. By tailoring PT to the specific bacterial strain, it was possible to achieve significant symptom relief and a substantial reduction in the bacterial load in the patient. This case demonstrated the unique ability of PT in the treatment of infections in areas like the prostate, where antibiotic penetration is limited. Research confirms the potential of PT to treat chronic antibiotic-resistant infections in anatomically challenging areas, providing an option where traditional treatments fail (Johri et al., 2021).

Additionally, Rostkowska et al. (2021) conducted a study in which PT was used in a kidney transplant patient with rUTIs caused by ESBL-producing *K. pneumoniae*, unresponsive to antibiotic treatments. The experimental PT successfully controlled the infection after the nephrectomy of infected kidney. The patient experienced no side effects from PT and the infection was resolved. This case underscores the feasibility of PT in patients with limited antibiotic options, particularly those transplant recipients undergoing immunosuppressive treatments (Rostkowska et al., 2021).

In turn, Corbellino et al. (2020) successfully utilized personalized PT to treat a persistent infection caused by carbapenemase-producing *K. pneumoniae*. The patient who suffered from chronic kidney failure and had a permanent ureteral stent, experienced rUTIs despite multiple treatments with ceftazidime-avibactam. After developing a custom phage formulation, the patient underwent a three-week PT course, which effectively eradicated the MDR *K. pneumoniae* strain. This landmark case highlighted the potential of personalized PT as an effective treatment for infections that do not respond to conventional antibiotic therapies, providing key evidence of the efficacy of PT in combating MDR bacteria (Corbellino et al., 2020).

Available literature data indicate that PT may be a promising alternative treatment for UTIs caused by antibiotic-resistant bacteria. However, more well-designed clinical trials are needed to establish the safety and clinical efficacy of PT as a therapeutic intervention for UTIs (Al-Anany et al., 2023).

## Phage therapy in urology: safety and efficacy

Phage-based therapy shows promise as an alternative to antibiotics for the treatment of MDR bacterial infections, offering high tolerability and minimal disruption of the microbiome (Mishra et al., 2024). Uyttebroek et al. (2022) reported a 7% adverse event rate in patients receiving PT versus 15% in controls, with clinical improvement in 79% and bacterial eradication in 87% across studies, though most cases involved antibiotics. In turn, the study conducted by Al-Anany et al. (2023) allowed to observe bacterial eradication in 76% after administration of phages and symptom improvement in 97% of urological cases (although antibiotics were discounted only in 22%). Moreover, a single randomized trial in urology showed an 18% success rate for PT compared to 35% with antibiotics (Leitner et al., 2021).

Despite its promise, safety concerns remain a critical consideration for PT in human use. One advantage of this therapy is its specificity, which minimizes harm to beneficial microbiota compared to broad-spectrum antibiotics (Sweeney et al., 2018). However, the potential of phages to transfer antibiotic resistance genes between bacteria raises concerns about their role in spreading resistant strains. This phenomenon, known as horizontal gene transfer, necessitates extensive research to determine its prevalence and implications in clinical settings (Gunathilaka et al., 2017).

Another safety challenge comes from the release of endotoxins during phage-mediated bacterial lysis. As phages destroy their bacterial targets, endotoxins, a component of the bacterial cell wall are released into the bloodstream. In mild cases, this can cause fever, but in severe instances, it could lead to systemic inflammatory responses, septic shock, or even organ failure (Taati Moghadam et al., 2020). This risk is particularly concerning in infections with a high bacterial load, where rapid lysis could overwhelm the body’s capacity to manage the endotoxin surge (LaVergne et al., 2018; Ujmajuridze et al., 2018).

Immune system activation is another factor. Phages can stimulate both innate and adaptive immune responses, leading to the production of antibodies and inflammatory cytokines (Lin et al., 2020). While most immune reactions are mild and transient, high doses of phages or prolonged exposure may increase the risk of significant inflammation or hypersensitivity. These reactions are rare but warrant consideration, particularly in immunocompromised patients or those with pre-existing inflammatory conditions (Taati Moghadam et al., 2020).

In addition, contamination during phage preparation is a serious safety concern. Phage preparations may contain bacterial debris, DNA, or residual endotoxins if purification protocols are inadequate (Yangyanqiu and Shuwen, 2022; Van Belleghem et al., 2017). These contaminants can exacerbate immune responses or introduce unintended risks. Rigorous purification processes and standardized production protocols are essential to address this (Ferry et al., 2018). Ongoing efforts to establish regulatory guidelines for phage manufacturing will play a critical role in ensuring the consistency and safety of therapeutic products.

In conclusion, while phage-based therapy holds significant potential, particularly against antibiotic-resistant infections, its safety profile must be carefully managed (Chung et al., 2023). Addressing risks such as endotoxin release, immune reactions, and contamination through rigorous selection, purification, and dosage protocols is essential to ensure its effectiveness and safety for human use.

## Discussion

Phages, as naturally occurring bacterial viruses, have been used for decades as stand-alone therapeutic agents (local and systemic treatment) or in combination with antibiotics. As bacterial parasites, phages kill only bacteria without a negative impact on eukaryotic cells, in which they do not multiply (Principi et al., 2019; Zalewska-Piątek, 2023). Due to the rapid increase in the number of MDR pathogens worldwide, mainly in the last decade combined with the decline in the development and production of new antibiotics, there has been an increased interest in phages and phage-based therapies against a wide range of diseases (Murray et al., 2022). The widespread use of phages is also dictated by the fact that in the last 20 years, the U.S. FDA has approved only two new classes of antibiotics against Gram-positive bacteria (Shi et al., 2023).

The action of phages is unique and different in relation to currently used and known pharmaceuticals and antibacterial agents (Parracho et al., 2012). Therefore, it is necessary to have a standardized protocol for their use in the treatment of infectious diseases caused by MDR bacteria. In the case of phage preparations, their purity, safety, sterility and stability are important. Therefore, the methods of obtaining therapeutic phage preparations should be continuously improved in order to reduce the number of impurities and increase the possibility of undesirable side effects during therapy (Chung et al., 2023).

However, complete, well-controlled clinical trials are still needed to analyze the efficacy and safety of PT against bacterial infections. Considering the regulatory improvements regarding the medical application and commercialization of phage preparations, detailed studies on phage genetics, different forms of phage-based therapy (phage cocktails, phage-antibiotic combinations, phage enzymes) or personalized treatment, indicate a dynamic future development of PT (Zalewska-Piątek, 2023; Mishra et al., 2024).

## Abbreviations

UTIs, urinary tract infections; AMR, antimicrobial resistance; IBCs, biofilm-like intracellular bacterial communities; QIRs, quiescent intracellular reservoirs; PT, phage therapy; MDR, multidrug-resistant; CAUTIs, catheter-associated UTIs; rUTIs, recurrent UTIs; UPECs, uropathogenic *E. coli*; WWII, World War II; FDA, Food and Drug Administration; EMA, the European Medicines Agency, INDs, Investigational New Drugs; MRSA, methicillin-resistant *S. aureus*; VRE, vancomycin-resistant *Enterococci*; ESBL, extended-spectrum β-lactamase; XDR, extensively drug-resistant; CBP, chronic bacterial prostatitis.
